# Endoplasmic reticulum stress associates with the development of intervertebral disc degeneration

**DOI:** 10.3389/fendo.2022.1094394

**Published:** 2023-01-12

**Authors:** Jishang Huang, Qingluo Zhou, Qun Ren, Liliang Luo, Guanglin Ji, Tiansheng Zheng

**Affiliations:** ^1^ Department of Orthopedics, First Affiliated Hospital of Gannan Medical University, Ganzhou, China; ^2^ College of Pharmacy, Gannan Medical University, Ganzhou, China; ^3^ Department of Orthopedics, Shangyou Hospital of traditional Chinese Medicine, Ganzhou, China

**Keywords:** intervertebral disc degeneration, endoplasmic reticulum stress, apoptosis, PERK, IRE1α, ATF6

## Abstract

Endoplasmic reticulum (ER) is an important player in various intracellular signaling pathways that regulate cellular functions in many diseases. Intervertebral disc degeneration (IDD), an age-related degenerative disease, is one of the main clinical causes of low back pain. Although the pathological development of IDD is far from being fully elucidated, many studies have been shown that ER stress (ERS) is involved in IDD development and regulates various processes, such as inflammation, cellular senescence and apoptosis, excessive mechanical loading, metabolic disturbances, oxidative stress, calcium homeostasis imbalance, and extracellular matrix (ECM) dysregulation. This review summarizes the formation of ERS and the potential link between ERS and IDD development. ERS can be a promising new therapeutic target for the clinical management of IDD.

## 1 Introduction

In recent decades, low back pain has been regarded as the main cause of disability, which brings heavy mental pressure and economic burden on patients (1, 2). An epidemiological survey finds that the direct medical cost of low back pain is as high as $100 billion in the United States, without considering the indirect costs caused by the decline of patients’ labor capacity (3). It is well known that intervertebral disc degeneration (IDD) is one of the leading causes of low back pain, and the prevalence of IDD increases dramatically with aging. Studies have been shown that more than 90% of people over 50 have IDD (4). Although the etiology and mechanism of IDD are still being explored, a large number of studies indicate that the pathological development of IDD can be attributed to various factors, such as excessive mechanical load, insufficient nutritional supply, metabolic disorders, immune mechanism, and inflammation.

IDD is a pathological process in which the intervertebral disc (IVD) degenerates with aging, resulting from a complex interaction between environmental and genetic factors. Currently, many therapeutic strategies are available for the treatment of IDD, mainly including drug treatment and surgical therapy. However, most of these strategies have limitations (5). Early drug treatment can only slightly reduce patients’ suffering, and very few drugs are approved against IDD pathogenesis and for disc protection. Arthroplasty with artificial disc replacement for surgical treatment has been introduced with the notion that it may decrease the risk of adjacent segment disease (6). Although the degenerated IVD can be removed surgically, some patients after surgical therapy may suffer from long-term post-spine syndromes and lumbar pain due to the destruction of the original biological structure (7). In addition, a discectomy may accelerate the progress of disc degeneration (8).

The degenerative process of IVD, demonstrated by the pathological changes at the molecular, cellular, and tissue levels, is orchestrated by a variety of signaling pathways. Endoplasmic reticulum (ER) plays an important role in maintaining the normal physiological structure and exerting physiological functions in IVD. Endoplasmic reticulum stress (ERS) can be induced by abnormal accumulation of unfolded or misfolded proteins and be involved in the mechanism of musculoskeletal disorders, including IDD (9–12), osteoarthritis (13), osteoporosis (14), rheumatoid arthritis (15), and muscular dystrophy (16). Particularly, some studies have been shown that ERS is associated with the pathogenesis of IDD by mediating oxidative stress, calcium homeostasis imbalance, inflammatory responses, excessive mechanical loading, and metabolic disturbance. In this review, we will specifically focus on the activation of ERS and its role in the pathological development of IDD, as well as discussion on the potential of reducing ERS as a therapeutic target against IDD.

## 2 The pathological development of IDD

Anatomically, IVD is a cylindrical avascular connective tissue located between two adjacent vertebral bodies. It is composed of a central nucleus pulposus (NP), a peripheral annulus fibrosus (AF), and a cartilage endplate (CEP) and plays a vital role in maintaining normal spine height and physiological curvature and coordinating spine movement. The extracellular matrix (ECM) provides a microenvironment for IVD cells and it maintains a dynamic balance between synthesis and degradation under normal conditions and ensures the physiological functions of IVD. Proteoglycans and collagens are the main biological macromolecules in ECM. Once the molecular dysregulation and structural abnormalities in ECM occur, the degradation and loss of proteoglycan will be triggered and the earliest pathological changes are initiated during the pathological development of IDD. It has been demonstrated IDD development is influenced by various factors, such as excessive mechanical loading, hypoxia, metabolic disorders, immune dysregulation, and inflammation (17–23). The pathological changes include a progressive decrease in nutrient supply, alterations in ECM composition, and cell apoptosis in IVD. The imbalance between the anabolism and catabolism of IVD cells can be reflected by the changes in ECM components, including the decrease of proteoglycan content and the replacement of type II by type I collagen fiber. More importantly, studies have been shown that the degenerated IVD cells are often accompanied by abnormal gene and protein expression of matrix proteases, such as MMPs and a disintegrin and metalloproteinase with thrombospondin motifs (ADAMTSs), through the possible mechanisms in mediating oxidative stress, inflammatory responses, and acidic metabolites (24–26).

## 3 Activation of ERS contributes to IDD pathological development

### 3.1 ERS and the unfolded protein responses

ER is a three-dimensional tubular network structure composed of small tubes, vesicles, and flat sacs. As the largest organelle in eukaryotic cells, ER is involved in the folding of proteins, the post-translational modification of various proteins, and a reservoir for intracellular calcium ions (27). Almost all proteins on the endomembrane system are synthesized in ER membrane-bound ribosomes and followed by post-translational folding in the ER lumen (28). According to the metabolic needs, cells regulate ER’s functions to ensure the accuracy of protein folding. Although the folding activity in the ER is well maintained, misfolding of nascent peptide chains and defects in protein assembly are unavoidable. Occasionally, some generated residues are recognized by the transporters and polyubiquitinated in the cytoplasm by the proteases for degradation, which is called ER-associated degradation (ERAD) (29).

ERS occurs when the protein processing is blocked or the misfolded proteins accumulate in the ER (30). The stability of ER internal environment is a prerequisite for the realization of functions. When ER is overwhelmed, there will be a large accumulation of unfolded or misfolded proteins. When the accumulated proteins exceed the folding and degradation capacity of ER, ERS will be initiated. Many factors are involved in the induction of ERS, such as inflammatory responses, excessive mechanical loading, and metabolic disorders.

When ER is stressed, cells sense the accumulation of the unfolded or misfolded proteins and restore ER homeostasis through three signaling pathways (31). This regulation is called unfolded protein response (UPR) (32), which is an adaptive response against the accumulation of the unfolded or misfolded proteins. Cells relieve ERS by reducing protein synthesis, promoting protein degradation and efflux, and inducing the synthesis of molecular chaperones to enhance the protein folding capacity. Interestingly, UPR also increases the area of the ER membrane by increasing the expression of lipid metabolism-related genes, thereby expanding the ER capacity (33, 34). Ultimately, if ERS is overwhelmed or prolonged beyond the cell’s adaptive capacity, UPR will trigger cell apoptosis (31, 35).

**Figure 1:**
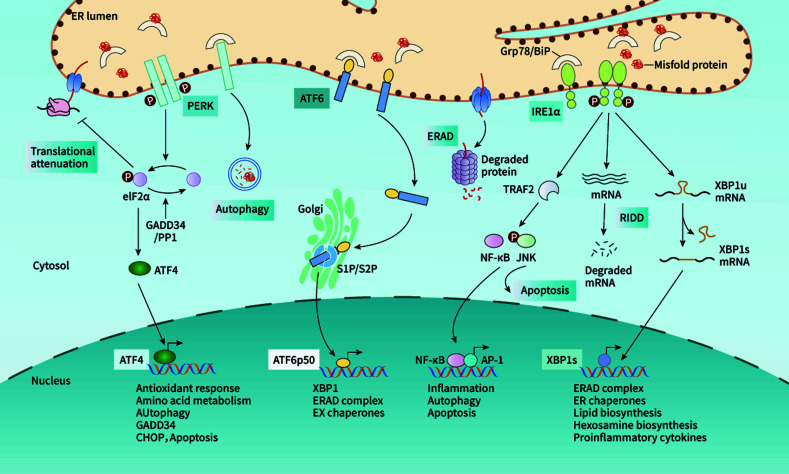
The UPR regulates the protein folding processes by activating three signaling pathways. Under stress, the release of GRP78 induces activation of PERK, which phosphorylates eIF2α and then upregulates the expression of ATF4. As a transcriptional factor, ATF4 enters the nucleus and mediates the expression of downstream factors. Similarly, IRE1α triggers the splicing activity to produce spliced XBP1, which is also a transcriptional factor in regulating the target gene expression. In addition, IRE1α may also initiate cell apoptosis by activating TRAF2/JNK signaling. ATF6 is transported to Golgi, where it is cleaved and activated.

The UPR regulates the folding processes of proteins through three ER-localized transmembrane proteins, protein kinase R (PKR)-like endoplasmic reticulum kinase (PERK), inositol-requiring enzyme-1α (IRE1α), and activating transcription factor 6 (ATF6) (**Figure 1**) (33, 36). Normally, the N-termini of these ER transmembrane proteins are tightly bound to the ER molecular chaperone GRP78/BiP to maintain their inactive forms. However, when the unfolded protein accumulates in the ER, GRP78/BiP is ectopically released, and phosphorylation of these ER transmembrane proteins results from a conformational change.

PERK is an ER-resident Ser/Thr protein kinase (37) and its catalytic domain shares high homology with the kinases in the eukaryotic initiation factor 2α (eIF2α) family. Once GRP78 is released, PERK is phosphorylated and its kinase domain is activated (37, 38). Activated PERK phosphorylates the protein translation initiation factor eIF2α, which up regulates the expression of ATF4. Additionally, ATF4 may drive the expression of the downstream target gene CHOP and control the encoding of apoptosis-related genes (39). IRE1α is a bifunctional transmembrane kinase/endoribonuclease (RNase) to exhibit dual enzymatic activities. When GRP78 is released, IRE1α dimerizes and stimulates autophosphorylation, inducing a conformational change to activate the ribonuclease domain. IRE1α determines cell fate through unconventional splicing of mRNA and regulated IRE1α-dependent decay (RIDD). When the UPR occurs, the XBP1 mRNA becomes the direct target of the endonuclease action of IRE1α. The spliced XBP-1 can promote the expression of UPR target molecules containing ERS response elements, such as GRP78. IRE1α initiates RIDD for the UPR target genes selectively to enhance ERS intensity (40). Once the intensity reaches the threshold, RIDD initiates mitochondria-dependent apoptosis (41–46). In addition, activated IRE1α also induces the phosphorylation of c-Jun N-terminal kinase (JNK), which leads to cell apoptosis (42). Compared with PERK and IRE1α, ATF6 turns on a distinct protein activation mechanism after dissociation from GRP78. When the unfolded protein accumulates, GRP78 dissociates from ATF6 and loses its inhibitory effect on Golgi localization signal (GLS), leading to the translocation of ATF6 to the Golgi apparatus. The transmembrane fragment of ATF6 is cleaved by a resident protease at the juxtamembrane site of the Golgi apparatus (47). The fragment activated by ATF6 cleavage can enter the nucleus to upregulate the expression of UPR molecules, such as GRP78 and GRP94. Meanwhile, activated ATF6 can also promote the expression of XBP1 and CHOP and work together with ATF4 to activate the ERAD pathway, alleviating ERS (48, 49).

### 3.2 The role of ERS in the pathological changes of IVD cells

IVD is a high loading site for protein synthesis, and ER is more likely to withstand the pressure of protein synthesis and folding. Numerous studies have demonstrated that ERS plays an important role in IVD degeneration (11, 50, 51). It has been reported that the expression of ERS-related markers is significantly increased in a rat IDD model, which is constructed by surgically removing the sacrospinous muscle, spinous process, supraspinous ligament, interspinous ligament, and the posterolateral part of the bilateral facet joints of the lumbar spine (5, 52). Interestingly, when the UPR branch inhibitor GSK2606414 is added or the transcriptional expression of ERS pathway target proteins is knocked down by lentiviral transfection, the apoptosis rate of FBS starvation-treated AF cells is significantly reduced. Increased AF cell apoptosis can accelerate the progression of IDD, and GSK2606414 treatment may ameliorate IDD development (51). These studies strongly demonstrate the involvement of ERS in the development of IDD.

#### 3.2.1 ERS induces cell apoptosis during IDD development

**Figure 2:**
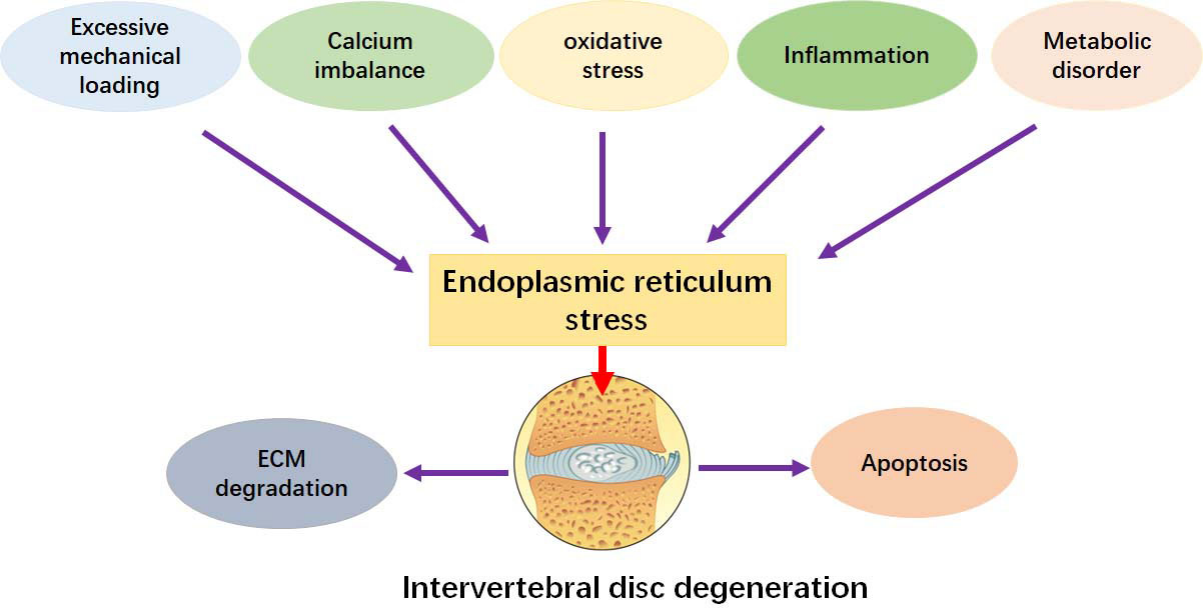
ERS promotes the development of IDD. ERS can be activated by inflammation, apoptosis, senescence, oxidative stress, mechanical pressure, calcium dyshomeostasis, and metabolic disorder. These factors can facilitate the pathological changes by stimulating ERS.

The epidemiological study shows that the proportion of middle-aged and elderly patients with IDD is significantly higher than that of young people (4). Through transmission electron microscopy and tissue section observation, the apoptosis of human degenerated NP cells and endplate chondrocytes also increased significantly (51). Another key pathogenic factor for the induction of IDD is cellular senescence. NP cells can be induced to senesce under a simulated acidic microenvironment *in vitro*, and activation of UPR signaling can compromise the process (26). Cell death triggered by activation of apoptotic signals in IVD cells is the main cause of cells loss and ECM degradation in IDD (**Figure 2**).

IDD-related apoptosis pathways include death receptor pathway, mitochondrial pathway, and ERS pathway. In the degenerated IVD tissues, the ERS levels and apoptosis rates increase, and ERS promotes NP cell apoptosis and IDD progression. Specifically, IRE1α inhibits the transcription of microRNA and initiates mitochondria-dependent IVD apoptosis (45). Inhibition of IRE1α activity significantly slows the degeneration of NP cells and the progression of IDD *in vivo* (53). Similarly, activation of PERK/eIF2α pathway at the early stage of the UPR can inhibit the apoptosis of NP cells by activating autophagy. However, CHOP acts as the main transcription factor linking ERS to apoptosis. The expression of CHOP and its downstream effectors caspase-12 and GADD34 in IVD cells can be significantly upregulated (54, 55). In one study, TNF-α promotes NP cells apoptosis by enhancing the expression of CHOP (55). Apoptosis activation is closely related to the expression of CHOP (56). Mild ERS has a protective effect on IVD (57, 58), and severe ERS may be related to cell senescence and apoptosis, which participate in the development of IDD. Collectively, ERS-induced cell senescence and apoptosis play an important role in the induction of IDD pathological features. However, it is still unclear what threshold of ERS will lead to the switch from protection to injury.

#### 3.2.2 ERS affects ECM metabolism during IDD development

The constituents of ECM mainly include water, collagen, proteoglycan, elastin, glycosaminoglycan, and glycoprotein. Various elements cross-link into a coordinated functional network (59, 60), which is involved in the metabolism regulation, compression cushion, and microenvironmental homeostasis. ECM acts as a repository of ER-secreted growth factors and regulates the metabolism of IVD through the interaction of matrix components with various growth factors (61–63). Current evidence shows (53, 64–67) that a series of factors, including mechanical stress, inflammation, and oxidative stress, promotes the degradation of ECM. These may induce persistent overloading of ER or dysfunction of ER-resident molecular chaperones, contributing to the main pathological characteristics of IDD (68–70).

IDD development is associated with an ERS-mediated process of ECM metabolism that ultimately leads to pathological changes in the structures and functions of IVD (60, 71). Consistently, another study shows that more dilated and abundant ER and higher UPR target gene expression are observed in the degenerated NP cells isolated from IDD (12). Impaired anabolism of ECM has been associated with the development of IDD (53, 64, 72). Zhen Lin et al. reports that eicosapentaenoic acid can inhibit the mRNA and protein expression of ERS markers, such as GRP78, ATF4, and CHOP, and promote the synthesis of ECM components collagen II and aggrecan, indicating potential crosstalk between ERS and ECM metabolism in IDD (73). Previous studies identify that AGEs accumulation in IVD acts as an essential risk factor associating with cell apoptosis and impeding ECM metabolism *via* ERS (74). Furthermore, suppression of AGEs-induced metabolism impairment and cell apoptosis after ERS inhibition has been observed (74). Inflammation also impairs the metabolic activity of collagen and proteoglycans (53, 64).

Excessive activation of ECM catabolism promotes ERS-induced IDD progression (75). It has been demonstrated the presence of aggrecan catabolic products in both normal and degenerated human IVD tissues activates ERS with different levels (76). Consistently, the levels of biglycan and decorin, the degradation products of native glycanated forms, increase in rat NP cells with persistent activation of ERS (71, 72). Additionally, NP cells cultured under hypoxia show higher ERS and ADAMTSs expression, which contribute to ECM degradation (75, 76). Accumulation of ECM catabolic products may in turn lead to a stress-related response involving increased expression of Grp78 and protein disulfide isomerase, which further deteriorate the microenvironment of IVD (77–79). The imbalanced gene expression of MMPs and ADAMTSs is the important risk factor to disrupt ECM homeostasis in IDD (80, 81). Recent evidence shows that ERS acts as a key regulatory mechanism to regulate the expression of MMPs and their inhibitors (53, 72, 82). Specifically, cholesterol increases the gene and protein expression of MMPs and activates ERS in NP cells by stimulating the maturation of SREBP1 (72). Inhibition of IRE1 activity prevents the gene and protein expression of MMPs and ADAMTSs in IL-1β-treated NP cells (53).

Collectively, both metabolism balance and IVD structure can be negatively affected by ERS, as indicated by disorganized disc morphology, disruption of the lamellar collagen architecture, and reduction of collagen fibers and collagen (10, 51, 83, 84). However, the specific mechanisms of ERS in affecting IVD structure by interfering with metabolism still needs to be further studied.

### 3.3 The possible mechanisms of ERS in mediating IDD development

#### 3.3.1 The interaction between ERS and oxidative stress during the development of IDD

It has been reported that the degeneration of IVD is associated with an increased level of oxidative stress (65). H_2_O_2_ can promote NP cell apoptosis by activating the ATF4/CHOP signaling pathway. Peroxynitrite, a potent oxidant, may cause cellular tyrosine nitrosylation, which has been considered a hallmark of ROS overproduction (85). Notably, the number of nitrotyrosine-positive cells in human NP tissues increases with the aggravation of IDD (86, 87). Furthermore, oxidative stress has been implicated in matrix degradation, inflammation (88), and the reduction of cell viability and functions in the diagnostic device microenvironment *in vitro* (89, 90). Specifically, excessive ROS modifies matrix protein expression, causing oxidative damage to ECM, nutrient metabolism dysregulation, and mechanical impairment in IVD (67, 91). ERS may produce oxidative stress through the formation of disulfide bonds, activation of NADPH oxidase 4, and ER flavoprotein, promoting the progression of IDD (92–94). Oxidative stress can be a linker between ERS and IDD pathological features (95, 96). Some potential mechanisms might be implicated in the regulation of ERS-related oxidative stress in IDD development. Firstly, increased levels of ROS trigger calcium release from the ER and increase the sensitivity of calcium channels on the ER membrane, thereby delivering positive feedback for enhancing ERS (97, 98). Secondly, ROS generation impairs the ubiquitin-proteasome pathway and impedes the degradation of unfolded proteins (99–101). Finally, ROS also enforces ERS by mediating the formation of disulfide bonds and affecting the folding of proteins (92).

#### 3.3.2 The interaction between ERS and imbalanced calcium homeostasis during the development of IDD

Oxidative stress may contribute to the imbalance of calcium homeostasis. In addition, imbalance of calcium homeostasis and oxidative stress are the two factors involved in the progression of IDD (**Figure 2**). Ca^2+^ is an important messenger for intracellular signal transmission, and ER is responsible for intracellular Ca^2+^ storage. Under the condition of severe or prolonged ER dysfunction, Ca^2+^ is released from ER into mitochondria and subsequently triggers a series of apoptotic signal transduction pathways (102). This translocation is regulated by the IP3R-GRP75-VDAC1 signaling, and the imbalanced calcium homeostasis is involved in the development of IDD by initiating NP apoptosis (67). Advanced glycation end products (AGEs) have been reported to elevate intracellular calcium, deplete Ca^2+^ in the ER lumen, impair Ca^2+^ homeostasis, and promote NP apoptosis through ERS in a concentration- and time-dependent manner, exacerbating IDD development in rats (74).

#### 3.3.3 The interaction between ERS and inflammation during the development of IDD

Oxidative stress can interact with inflammation reciprocally. Inflammation is also one of the important factors involved in the development of IDD (**Figure 2**) (57, 103). Several studies have been shown that the expression of TNF-α and IL-1β in the degenerative IVD is significantly higher than that in the non-degenerative IVD (64, 104). Proinflammatory cytokines IL-1β, IL-6, and TNF-α are the key factors to activate ERS signaling and promote IVD cell apoptosis, leading to the development of IDD. In addition, the expression levels of TNF-α and IL-1β are positively correlated with the severity of IDD (64, 105–108). In IL-1β-treated human NP cells, a decrease in the expression of type II collagen and proteoglycan and an increase in the expression of TNF-α (mRNA), IL-6 (mRNA), MMP-13 (mRNA), GRP78 (mRNA and protein), and CHOP (mRNA and protein) are observed (79). In addition, in human NP cells co-stimulated with 5 ng/ml TNF-α and 1 ng/ml IL-1 β for 12 hours, the proliferation and anabolism are inhibited and the expression of PERK and IRE1 is increased. Inversely, knockdown of PERK and IRE1 increases the anabolism of NP cells. However, the expression of ATF6 is not significantly changed (12). Another study shows that IL-1β activates the protein expression of ER stress markers GRP78 and CHOP in human primary IVD cells (109).

It has been shown that upregulation of ERS and UPR signaling may compromise TNF-α-induced apoptosis in rat NP cells at an early stage and promote cell proliferation (110). Later, it is reported that PERK/eIF2α activation-induced autophagy protects TNF-α-treated NP cells from apoptosis (111). Another study reports that TNF-α (5 and 10 ng/mL) does not induce ER stress, while IL-1β (5 and 10 ng/mL) activates GRP78 expression but does not affect calcium mobilization (24). This discrepancy might be attributed to the different cell types and the reagent concentrations. The dual effects of PERK and IRE1-α on IVD cells may be associated with different stimuli under certain circumstances (12, 112, 113).

Evidence shows that ERS may synergy with inflammation to exacerbate IDD progression. ERS can activate the NLRP3 inflammasome to induce inflammatory responses through oxidative stress, calcium dysregulation, and NF-κB activation (114, 115). NOD-like receptors may mediate ERS-induced inflammation, and ERS-inducing agents trigger the production of the proinflammatory cytokine IL-6 in a NOD1/2-dependent manner (116, 117). ERS increases the mRNA expression of TNF-α and IL-6 in a NF-κB pathway-dependent manner in rat AF cells (51). Translational inhibition of IκB by PERK and upregulation of p65 phosphorylation by IRE1/XBP1 signaling may promote nuclear translocation of p65, effectively activating NF-κB signaling. However, the regulatory link between the UPR pathways and NF-κB signaling still needs to be elucidated.

#### 3.3.4 The interaction between ERS and excessive mechanical loading during the development of IDD

Excessive mechanical loading on IVD is another important factor in the development of IDD (66, 118) (**Figure 2**). Exposing AF cells to 18% deformation stress for 3 consecutive days can significantly increase the expression of caspase-12, caspase-3, and CHOP (119). Piezo1 is a mechanosensitive calcium channel, and it can be upregulated under the stimulation of mechanical tensile stress or shear stress. Excessive mechanical stress-activated Piezo1increases the senescence and apoptosis of NP cells, as well as the expression of GRP78 and CHOP (120). Cyclic stretching with a frequency of 0.5 Hz and an elongation rate of 20% has been reported to increase the expression of CHOP and GRP78 and induce apoptosis in rat AF cells (54, 56). Furthermore, the rate of apoptosis in CHOP shRNA-transfected AF cells was significantly reduced under the cyclic tensile stress (54). Excessive mechanical stress may transduce the signals into cells by mediating ERS, thereby regulating cell fate. In addition, some mechanosensitive channels, such as TRPV4, N-cadherin adhesions, and integrins, are involved in the regulation of IDD development. However, whether they are mediated by ERS is still unclear (121, 122).

#### 3.3.5 The interaction between ERS and metabolic disorder during the development of IDD

Metabolic disorder can stimulate IDD development (**Figure 2**). Abnormal glucose metabolism has been demonstrated to be a risk factor (123). A study shows that people with diabetes have a higher rate of IDD than healthy persons (124). In addition, high glucose also inhibits cell anabolism (119) and promotes IVD cells apoptosis (125). In AF cells, high glucose stimulates ERS and increases the gene and protein expression of CHOP, ATF6, and GRP78, which are the important factors in ERS. In addition, the apoptosis of AF cells is also increased (126). Due to the high blood glucose level, the chemotaxis of leukocytes is decreased, and the outcome of IDD surgery is often worse (127). Interestingly, glucose deprivation also induces apoptosis in NP cells, and this action can be inhibited by autophagy-mediated p-eIF2α/ATF4 pathway (128). Hypercholesterolemia may be a potential risk factor for IDD development. Specifically, cholesterol induces pyroptosis and matrix degradation through mSREBP1-driven ERS in a rat model with hypercholesterolemia. Similarly, the cholesterol-lowering drug atorvastatin can counteract these adverse effects of cholesterol on IVD (72). It has been reported that high concentrations of lactate promote the degradation of type II collagen and participate in the apoptosis regulation by activating caspase-3 expression and mediating autophagy (82). A study finds that activation of the acid-sensitive ion channel ASIC1a can promote the apoptosis of NP cells by regulating ERS (129). Interestingly, UPR-induced autophagy exerts an anti-degeneration effect on acid-treated rat NP cells (26).

## 4 ERS may become the potential therapeutic target to prevent IDD development

At present, there is no effective clinical strategies to cure IDD, due to the limited understanding of the molecular mechanisms. ERS has been demonstrated to play a key role in the development of IDD, and it has become the potential target for the therapeutic management of IDD. In recent years, some scholars have proposed the term “promoter of protein homeostasis” to define some small molecules with “chemical chaperone” activity (130, 131). For example, 4-phenylbutyric acid (4-Phenylbutyric acid, 4-PBA) and tauroursodeoxycholic acid (TUDCA) have been shown to reduce ERS by enhancing the folding ability of proteins (132). 4-PBA, a modified fatty acid, inhibits ERS and alleviates the progression of IDD. Consistently, 4-PBA can inhibit tension-induced IDD development, as indicated by reduced apoptosis, ameliorated ERS, and decreased ROS generation in AF cells (119). 4-PBA can also inhibit high glucose-induced apoptosis by inhibiting ERS in AF cells (126). TUDCA, a hydrophilic bile acid, has been reported to inhibit ERS-induced catabolism and reduce excessive mechanical compression-stimulated apoptosis in NP cells (133, 134). However, due to the poor selectivity, these chaperones usually require high concentrations to be effective, and it still needs further investigation to make the chemical chaperone-based therapeutics available for IDD management.

Inhibition of ATF4 expression can alleviate TNF-α-induced ROS production and apoptosis in NP cells (135). In addition, starvation-mediated TUNEL-positive frequency and apoptotic protein expression in AF cells can be significantly inhibited by PERK inhibitors (51). Despite the marked therapeutic efficacy, the on-target toxicity PERK inhibitors still needs for further investigation (136). In addition, ISRIB, a potent eIF2α inhibitor, significantly suppresses PTEN-deficient and MYC-overexpressing prostate cancer progression, extending the survival of tumor-bearing mice (137, 138). One study shows that pro-inflammatory cytokine intervention significantly upregulates the expression of IRE1-α and PERK, but does not change the expression of ATF6 (12). B-I09 has been proven a safe and selective IRE1α RNase inhibitor suitable for *in vivo* use. A preclinical study shows that treatment with B-I09 suppresses leukemic growth in mouse models with chronic lymphocytic leukemia without causing systemic toxicity (139). An RNase inhibitor of IRE1α can alleviate ERS-induced inflammatory responses in a mouse model with lipid metabolism disorder (140). These suggest that IRE1α pharmacological inhibitors may ameliorate the pathological development of IDD.

The Bip inducer sodium 2-propylvalerate (valproate) has been approved for clinical use in neuronal diseases and been in trials to protect pancreatic beta cells from ERS-induced apoptosis (141). BiP has been shown safe and effective anti-inflammatory and immunomodulatory properties in patients with rheumatoid arthritis (142). In addition, it has been reported that estrogen exhibits a critical role in protecting against the development of IDD in ovariectomized rats (143, 144). In addition, estrogen exhibits regulatory activity against ERS in different tissues (145, 146). However, whether estrogen is involved in inhibiting the pathogenesis of IDD by mediating ERS signaling deserves further study.

It is important to find that effective natural compounds from traditional Chinese medicine (TCM) play a role in regulating ERS and UPR pathways. This makes it possible to explore these natural compounds for the treatment of IDD. Berberine, an isoquinoline alkaloid isolated from *Coptis chinensis* and *Phellodendri* (147), has been reported to inhibit ERS-induced apoptosis *via* the IRE1/JNK pathway in NP cells (148). Quercetin, a bioflavonoid widely used in the treatment of the respiratory and cardiovascular diseases, has been shown to inhibit ERS and reduce chondrocyte degeneration and apoptosis by activating SIRT1/AMPK signaling pathway (149). Curcumin can significantly attenuate ERS-related chondrocyte apoptosis and improve the progression of osteoarthritis *in vivo* (150). Furthermore, 5-hydroxymethylfurfural and salidroside also exhibit the protective effects against ERS in various cell lines (151–153).

Exosomes are vesicle-like structures containing complex RNAs and/or proteins, which are widely involved in cell proliferation, migration, and other processes. Recently, it has been shown that exosomes have certain potentials in IDD treatment. Mesenchymal stem cell-derived exosomes can inhibit the apoptosis and calcification of endplate chondrocytes, and the mechanism may be related to the regulation of miR-31-5p/ATF6/ERS pathway (154). In addition, human urinary stem cell-derived exosomes (USCs-exos) have been also shown to significantly ameliorate ERS and inhibit apoptosis through AKT and ERK signaling pathways in NP cells, leading to attenuation of IDD development (155). Unfortunately, the specific mechanism by which exosomes mediate ERS signaling for the treatment of IDD has not yet been established.

## 5 Future perspective

The pathogenesis of IDD is complex and diverse. ER plays an important role in maintaining the functions of IVD and participating in the synthesis, transport, secretion, and degradation of ECM-related proteins (59, 156, 157). Current studies have been shown (18, 20–23) that various pathological factors, such as excessive mechanical loading, insufficient nutrient supply, metabolic disorders, immune mechanisms, and inflammation, lead to the chronic activation of ERS and promote the progression of IDD. However, ERS under physiological conditions has a protective effect on IVD cells (26, 110). Thus, there are still many problems to be solved in these areas. The dynamics of ERS activation remain unclear. For example, how does the process of accumulation of unfolded and misfolded proteins in the ER lumen affect ERS signaling activation and transmission? Does it include the chronological sequence of the three branch pathways of the UPR in response to ERS and of their respective roles? Do the mechanisms by which chronic noxious stimuli and acute severe factors trigger ERS and their effects act on IDD progression in the same way? How to determine the dividing line between whether ERS regulates life activities is beneficial or harmful? And how the ERS under physiological stress is regulated is still unknown. Many studies have been confirmed the benefits of inhibiting ERS in IDD. Whether ERS inhibitors have certain side effects on IVD or other parts of the body still needs to be further explored.

## 6 Conclusion

IDD has become a global public health problem. It is important to study the pathogenesis of IDD and explore effective prevention and treatment. Various potential mechanisms, including inflammatory responses, excessive mechanical loading, metabolic disturbance, oxidative stress, calcium homeostasis imbalance, and nutritional deficiencies, are included in the development of IDD. ERS plays an important role in the pathogenesis of IDD. However, targeting ERS for the treatment of IDD remains challenging. In recent years, many studies have been carried out to explore the relationship between ERS and IDD and investigate the intervention of ERS signaling to improve IDD. Clues are being enriched in ERS-targeted therapy for disc degeneration.

## Author contributions

TZ provided the idea of this paper. JH, QZ, QR, LL, and GJ collected the information and revised and finalized the paper. All authors approved the final paper.
